# A genome-wide spectrum of tandem repeat expansions in 338,963 humans

**DOI:** 10.1016/j.cell.2024.09.045

**Published:** 2024-10-04

**Authors:** Ya Cui, Wenbin Ye, Jason Sheng Li, Jingyi Jessica Li, Eric Vilain, Tamer Sallam, Wei Li

## Abstract

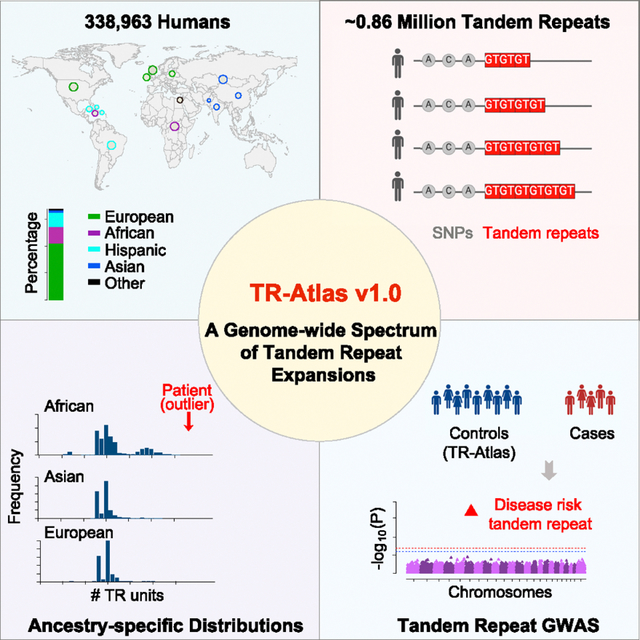

## Graphical Abstract (original)

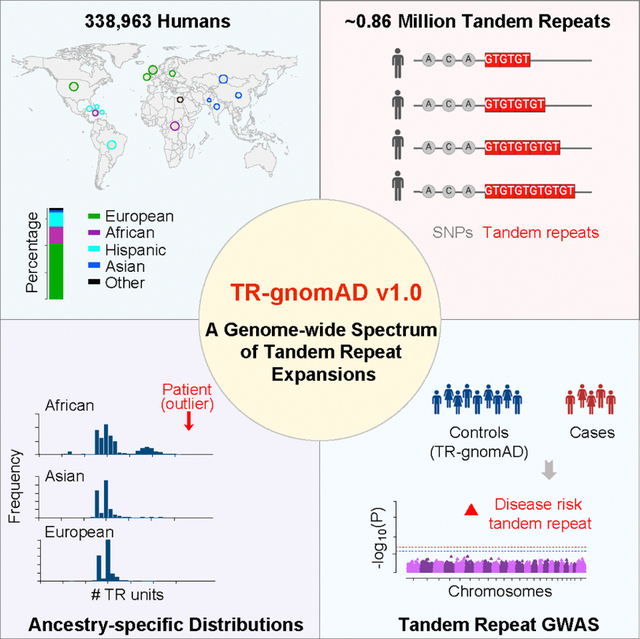

In the originally published version of this article, the tandem repeat reference resource was called TR-gnomAD. In order to avoid any confusion with the affiliation of the resource or where it was developed, we have changed the name to the Tandem Repeat Aggregation Atlas, or TR-Atlas, throughout the article and the supplemental files. We have also updated the link to this resource to https://wlcb.oit.uci.edu/TRatlas/. This change does not affect the contents or use of the resource in any way, nor does this affect any conclusions of the manuscript. The original article has now been corrected online to reflect this change.

